# Improved DNA Electrophoresis in Conditions Favoring Polyborates and Lewis Acid Complexation

**DOI:** 10.1371/journal.pone.0011318

**Published:** 2010-06-25

**Authors:** Hari Singhal, Yunzhao R. Ren, Scott E. Kern

**Affiliations:** 1 Department of Biomedical Engineering, The Johns Hopkins University and the Sidney Kimmel Comprehensive Center at Johns Hopkins, Baltimore, Maryland, United States of America; 2 Department of Oncology, The Johns Hopkins University and the Sidney Kimmel Comprehensive Center at Johns Hopkins, Baltimore, Maryland, United States of America; University of Poitiers, France

## Abstract

Spatial compression among the longer DNA fragments occurs during DNA electrophoresis in agarose and non-agarose gels when using certain ions in the conductive buffer, impairing the range of fragment sizes resolved well in a single gel. Substitutions using various polyhydroxyl anions supported the underlying phenomenon as the complexation of Lewis acids to DNA. We saw significant improvements using conditions (lithium borate 10 mM cations, pH 6.5) favoring the formation of borate polyanions and having lower conductance and Joule heating, delayed electrolyte exhaustion, faster electrophoretic run-speed, and sharper separation of DNA bands from 100bp to 12 kb in a single run.

## Introduction

DNA electrophoresis is arguably the most commonly performed molecular assay over the past fifty years. It is useful in restriction enzyme mapping, sequence analysis, confirmation of plasmid construction and PCR products, separation for Southern blotting and various other “downstream” techniques.

The technique was initially borrowed from protein and RNA techniques rather than primarily developing through design of optimized methods [1]. It generally employs suboptimal buffers having high ionic strength, conductance, and electric field strength. Excessive Joule heating limits the tolerable applied voltage and the speed of electrophoretic separation.

Recent modifications of DNA electrophoresis eliminated sodium EDTA and substituted alkali metal cations for tris. Lithium was preferred over other alkali metal cations for its large shell of hydration and low electrokinetic mobility, which provided lower conductance, improved tolerance for applied voltage, lower heat generation, and improved separation quality. Compared to tris-borate-EDTA (TBE), the alkali-metal ion media decreased the conductivity, lowered the final running temperature, and reduced the time for electrophoretic separation [2].

These systems are not yet optimal, as improvements from anion substitutions had not been much explored. Most anions at routine concentrations generate excessive current and heat and are oxidized at the anode, eroding buffer integrity. Also, for any particular anion, the properties of DNA electrophoresis vary depending on the range of DNA fragment lengths being separated [1]. Example, in common slab gels using 1% agarose under a homogeneous electric field, acetate anion resolves DNA bands in a range from 1 kb to 12 kb. Unfortunately, acetate gels have unacceptably poor separation and fuzzy bands in the range less than 500 bp, a tendency which is worse with the smaller fragments in this range. On the other hand, monoborate anion suitably resolves DNA bands below 1.5 kb but causes compression of DNA fragments above about 1.5 kb, a shortcoming that is progressively worse at greater lengths. Combining acetate with borate retains the deficiencies of both. The mechanism for compression is yet unclear, though it has been attributed to borate forming complexes with the DNA phosphodiester backbone [3]. This is supported by observations of lessened DNA compression in capillary electrophoresis using very high borate concentrations and also by the known complexes formed between hydroxylated boron species and the vicinal (adjacent) hydroxyl groups on carbohydrates [4]–[6]. Because these compressions affect gels formed of agarose (containing vicinal hydroxyl groups interacting with borates, [5], [6]) and non-agarose gels such as polyacrylamide (lacking such hydroxyls), the vicinal hydroxyls of the DNA molecule are suspected as a primary reactant when borate is not limiting (as in “submarine” gel electrophoresis and when gels are pre-run in excess buffer). Under conditions of a limiting content of borate (capillary electrophoresis), agarose is reported to compete for binding, rendering the agarose interaction dominant over the DNA interaction [6]. Ideally, one would prefer a corrective alternative applying both to the gel and the DNA.

Presently, drawbacks exist with various extended methods employed in agarose gels to resolve better the DNA bands. Field inversion electrophoresis requires special equipment and is time-consuming. Special gel matrices, such as polyacrylamide and chemically modified agarose, offer limited improvements for special situations by improving optical qualities, better dissipating Joule heating, focusing the DNA origin, and allowing shorter runs. Varying the gel agarose content achieves better resolution only for a limited range of fragment sizes, with the trade-off of worsening the resolution in other size ranges. Increasing the borate ion concentration was explored in capillary electrophoresis to saturate borate's cognate partners in complex formation [3]; it was shown to reduce the compression effect of TBE-based capillary separations, but lead to undesirable increases in conductance and Joule heating. Such approaches are impractical for slab gels as compared to capillary electrophoresis, since slab gels have relatively lesser surface area for heat dissipation.

### Guiding premises of our experiments

**Figure 1:**
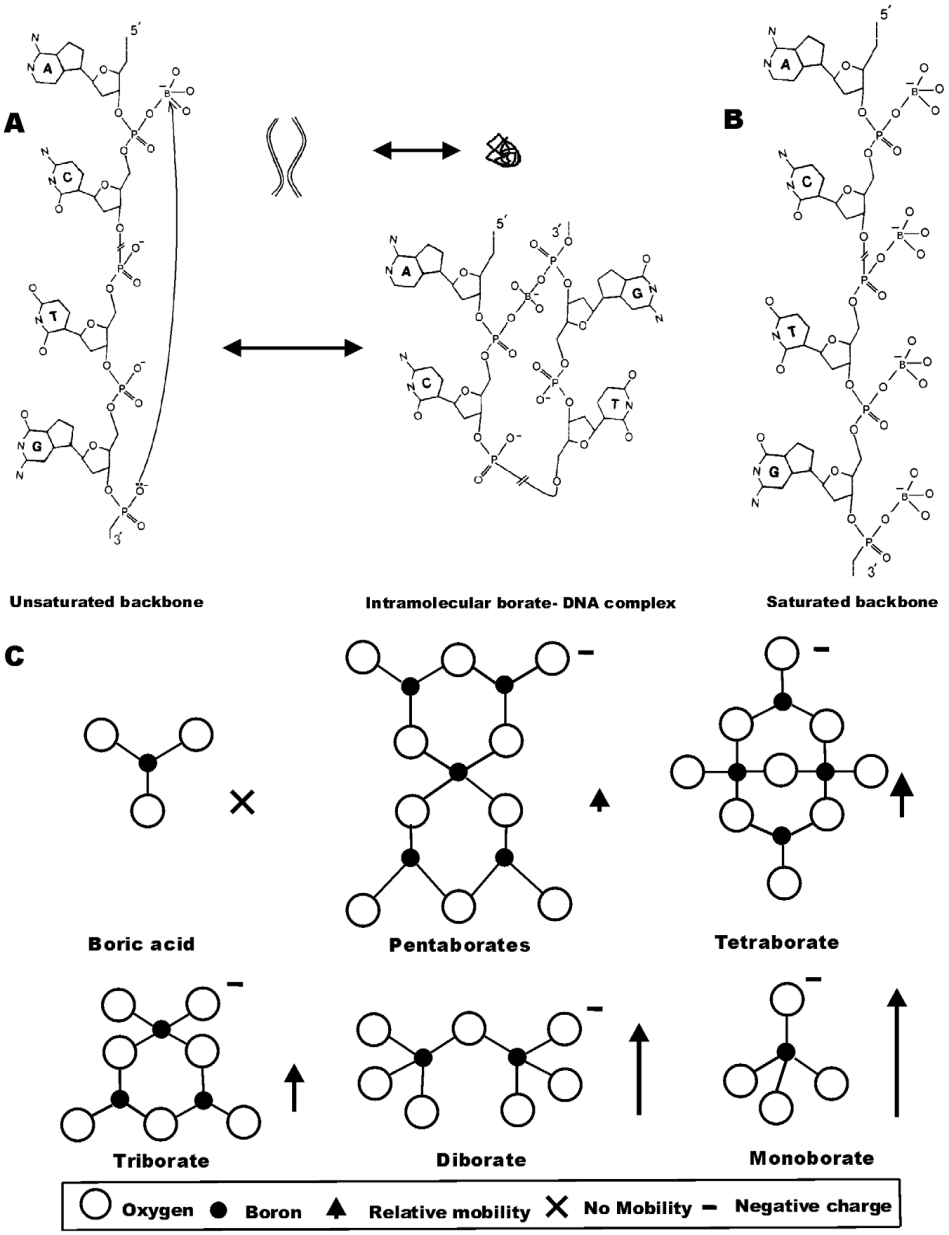
Guiding premise of the experiments and comparison of various polyborates. (a) Intra-molecular Lewis acid complex formation between boronic species and non-vicinal phosphate groups, located far apart on DNA backbone, leads to a lower gel apparent length. A structural diagram of a region of a single strand of duplex DNA and a smaller schematic cartoon of two duplex DNA fragments illustrate the molecular and topological consequences of complexation. Additional complexes, not depicted here, form due to hydroxyl groups in agarose matrices; in such gels, DNA-borate-agarose complexes are expected. (b) Saturation of DNA backbone with the boronic species prevents intra-molecular Lewis acid complexation. (c) Polyborates are formed at high boric acid concentrations. Mass-to-charge ratio increases and electromobility decreases (denoted by upward arrow) with the number of borates in each complex.

Hydroxyl groups in the phosphodiester backbone of DNA act as a general-type Lewis base and form Lewis acid complexes with the electron-deficient atoms in anionic compounds. That is to say, hydroxyl groups (Lewis acid bases) have electron pairs available to donate to electron-desiring chemical species (Lewis acids), lending the potential for new complexes to form between the DNA and other dissolved components of a solution. Relative to smaller DNA fragments, larger DNA fragments are more likely to participate in transient intra-molecular complexes between boron species and non-vicinal phosphate groups (perhaps located far apart along the DNA backbone), thus altering the DNA secondary structure so that it mimics a smaller apparent length during gel separation, figure 1A and 1B. Such contorted DNA fragments would migrate faster than linear ones, producing the compression of higher molecular weight DNA bands that is observed in these gels. Any improvements related to the interactions of DNA vicinal hydroxyl groups with other species (such as dissolved anions, which could be readily manipulated) were likely to similarly improve the interactions with analogous hydroxyl groups of agarose matrices (which would be more difficult to modify directly but were of high relevance in diverse gel formulations). We thus monitored improvements in DNA electrophoresis using agarose slab gels.

## Results

**Figure 2:**
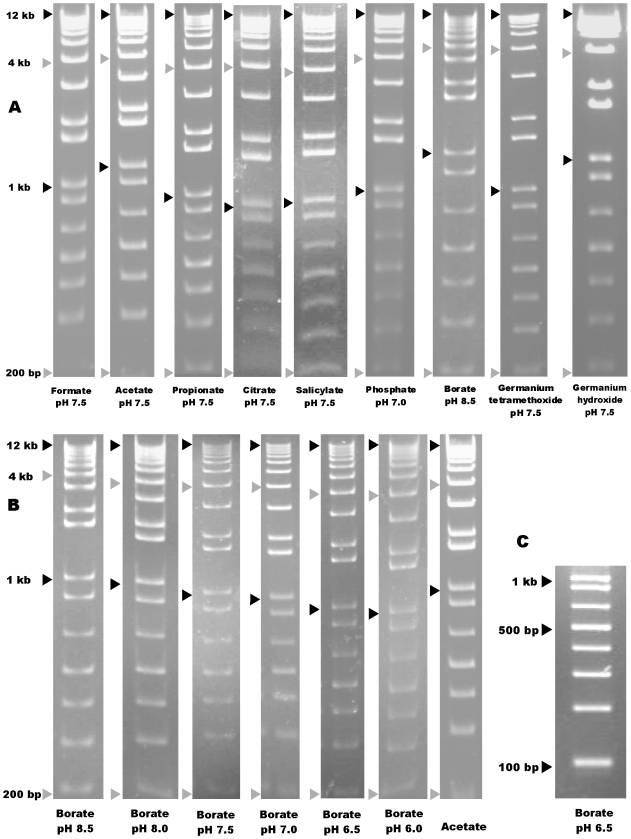
Evidence for Lewis acid complexation and improved borate electrophoresis at pH 6.5. DNA fragments of 4 kb, 1 kb and 200 bp are marked by arrowheads approximating the leading edges of the bands, and the 12 kb band is marked at the center of the lagging edge. The sizes of all fragments of the ladder were, in bp, 100, 200, 300, 400, 500, 650, 850, 1000, 1650, and 2000 through 12,000 in 1000-bp increments. (a) Electrophoretic separation for anions of differing Lewis acid character. (b) Separation using borates at different pHs, given a constant migration for the 200 bp band. (c) Separation (in 2% agarose) of bands from 100 bp to 1000 bp in 6.5 pH borate.

**Figure 3:**
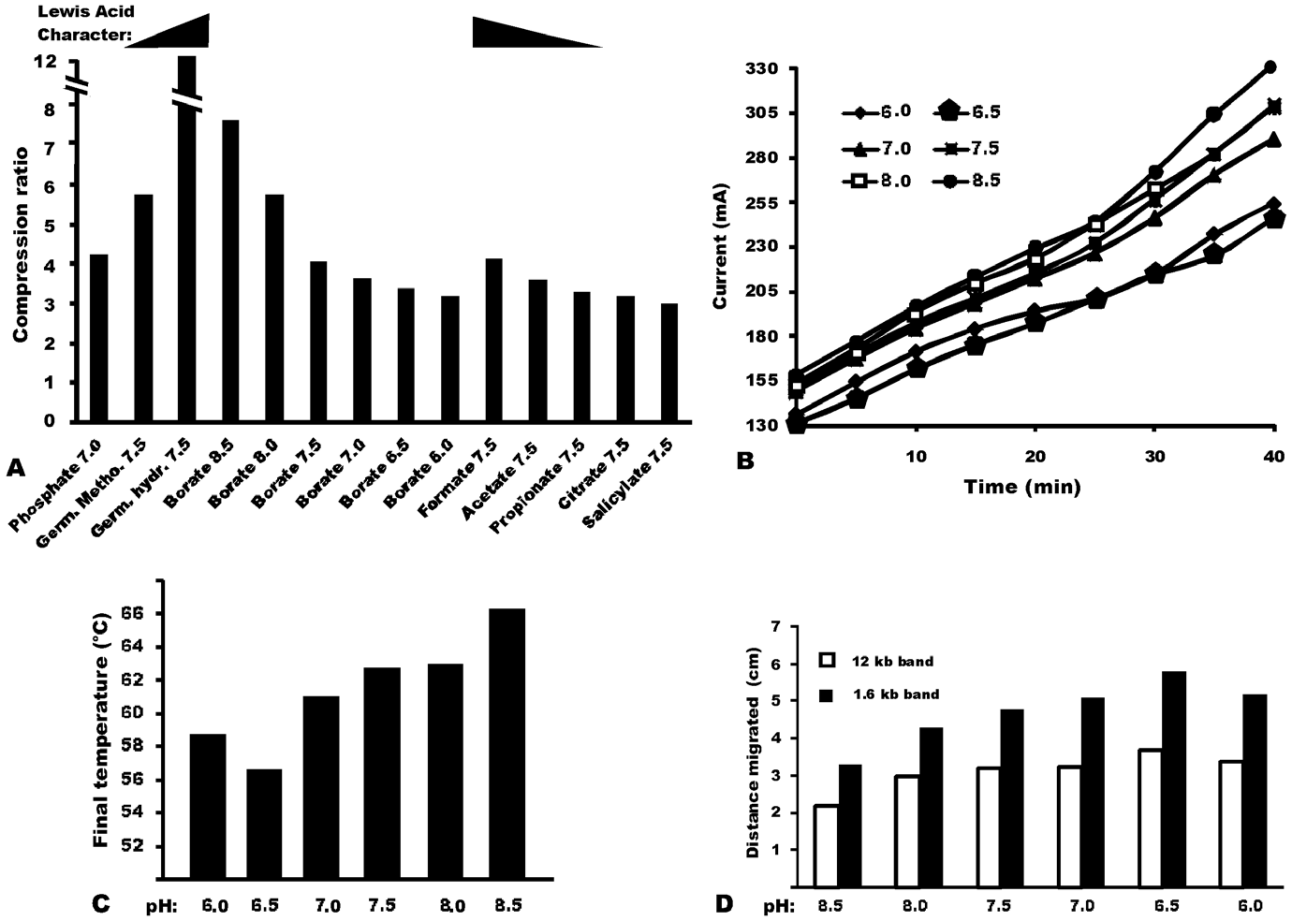
Compression ratios for anions tested and comparison of borate buffers at different pHs. (a) Compression ratios for various anions and borates at different pH. For the similar germanium compounds, the ramp above their data depicts that the hydroxide is expected to have the greater Lewis acid character. For the incremental linear chain carboxylic acid series (formate, acetate, propionate), the ramps above their data similarly depict that formate has the highest, and acetate the second-highest, expected Lewis acid character among this series. The ramps do not pertain to the other histogram data. (b) Conductivity of the buffer as a function of time, measured as current at constant voltage of 350 V. (c) Temperature in the anodic chamber as a function of time. (d) Electrophoretic run speed, represented as the distance traveled from the well by 12 kb and 1.6 kb bands in an 80 min run.

The relative distance between 12 kb band and mid-range bands, such as the 1.6 kb or 4 kb band, was greater for all the weak Lewis acids tested, compared to the various strong Lewis acids. Correspondingly, relative separation between 1 kb and 200 bp band was lesser for weak Lewis acids, figure 2A. The quality of separation was quantified by the compression ratios (defined in Materials and Methods), figure 3A. Higher ratios implied more compression.

In the hopes that the best of the electrophoretic qualities of borate (a hydroxide of boron) might be shared among the few soluble anionic hydroxides of neighboring elements in the periodic table, we explored commercially available oxidized compounds of carbon, germanium, and phosphorus at various values of pH. The nonorganic anions were attractive in theory due to their chemical stability (organic anions such as acetate will decompose oxidatively upon contact with the anode, while simple inorganic hydroxides in general are expected to remain unchanged). Formate had a higher compression ratio ( = 4.1), compared to acetate ( = 3.6), which in turn was higher than propionate ( = 3.3). Germanium tetramethoxide, Ge(OCH_3_)_4_, had improved separation with a compression ratio ( = 5.75) lower than germanium hydroxide, Ge(OH)_4_ ( = 12). Phosphate buffer had separations comparable to formate ( = 4.2), but unfortunately had moderately high conductivity and rapid Joule heating. The characteristics of most buffers did not change remarkably when explored at various values of pH or concentration (a subset of the data are presented in the figures). The exception was borate, and thus additional depth of study was provided to borate.

No increase in conductivity was observed on addition of boric acid to lower the pH of borate buffers. (This result might seem counter-intuitive. Boric acid, while electrically neutral in powdered form, can ionize to form conductive ions when dissolved. Yet, the overall borate anion concentration was obligatorily constrained; it must remain electrically balanced to the lithium cation concentration, which remained unchanged at 10 mM.) Remarkably, however, the borate buffer at pH 6.5 was observed to have 21% lower initial conductivity and 34% lower final conductivity than the same ionic concentrations at pH 8.5, figure 3B. In addition, lower conductivity lead to lower heat generation, the final temperature of pH 6.5 buffer after the run being 56°C, 10°C lower than the temperature for pH 8.5, figure 3C. Gels run in different pH borate buffers are presented in figure 2B. For comparison, an acetate gel is also included, which typically has minimal compression of higher molecular weight DNA. Compression ratios for borate buffers of different pH are compared in figure 3A. Despite lower heat generation, borate medium at pH 6.5 provided faster DNA electrokinetic speed. In an 80 minute gel run, relative to the well, the 12 kb band moved 3.4 cm at pH 6.5 compared to 2.2 cm at pH 8.5, and the 1.6 kb band moved 5.2 cm at pH 6.5 as compared to 3.3 cm at pH 8.5 (figure 3D). Sharp separation occurred for DNA fragments smaller than 500 bp even in pH 6.5 borate buffer, figure 2C.

No change in pH was observed during the electrophoresis run with lithium borate buffer. Borate buffers at all the different pHs studied had delayed buffer exhaustion of 100 minutes, a property making borate buffers appropriate for extended electrophoretic runs.

The pH 6.5 borate buffer was compatible with Qiagen, SpinX and Geneclean II gel elution procedures. Additionally, restriction digestion of the eluted DNA was successful (data not shown).

## Discussion

Compression of high molecular weight DNA bands above 4 kb was observed with all tested Lewis acid anions, namely borate, germanium hydroxide, and germanium tetramethoxide. In contrast, no electrophoretic compression of upper molecular weight bands was observed with anions lacking an electron-deficient atom (hence, being weak Lewis acids) such as acetate, salicylate, citrate, propionate and formate; such anions, however, did not produce acceptable resolution of smaller DNA fragments. Compression reappeared upon addition of boric acid to electrophoresis with acetate anions, additionally supporting Lewis acid complexation as the underlying cause (data not shown). Furthermore, compression due to borates disappeared at lower pH, presumably due to saturating the DNA backbone with an excess of neutral boronic species (such as monomeric boric acid, which carries no electric charge, or boric acid complexed to anionic borates, which would provide no additional net charge). This was achieved by decreasing the pH of lithium borate buffer to pH 6.5 upon addition of boric acid.

Due to a greater positive inductive effect of methoxy group than the hydroxyl group, germanium tetramethoxide is a weaker Lewis acid when compared to germanium hydroxide. Analogously we suppose that, due to the positive inductive effect of three methyl groups, propionate is a weaker Lewis acid than acetate, which in turn is weaker than formate, figure 3a. As additional support for the hypothesis, weaker Lewis acids had lower compression ratios than their respectively stronger Lewis acid counterparts.

Boron methoxide is a weaker Lewis acid as compared to borates and might be expected to show less compression than borates. However, the known hydrolysis of boron methoxide to borates and methanol precluded conducting unambiguous testing.

Owing to phosphate group being highly negative with three negative charges, Lewis acid complexation of phosphates with the phosphodiester backbone of DNA is presumably difficult due to electrostatic repulsion. Phosphate anion thus may have served as the theoretical, as well as the biologically intuitive, negative control. Despite having multiple oxygen groups and being an expected Lewis acid (an electron-deficient phosphorus atom connected to four oxygens), phosphate produced compression ratios similar to other weak Lewis acids. Teleologically, this fortunate property may have even favored phosphate polydiesters rather than other readily available soluble polydiesters for explorations of biological polynucleotides during evolution.

In summary, we noted that the Lewis acid character of the anion appeared to govern the formation of undesirable patterns, such as compression of higher-molecular weight DNA fragments. In exploring a solution using this clue, a wide variety of anions produced excessive conductivity and Joule heating at similar concentrations and run conditions. Borate anions, however, could be modified to improve electrophoresis, by lowering the pH through the addition of neutral boronic species. A high concentration of boric acid favors formation of polyborate anions, which have lower mobility due to higher mass-to-charge ratios [7], [8], figure 1C. Borate, in the neighborhood of pH 6.5, had several advantages over the conventional pH 8.0–8.5 media. Firstly, formation of polyborate anions lead to lower conductivity and lesser heat generation, and allowed higher running voltages. Secondly, it allowed uncompressed display of bands from less than 100 bp to greater than 12 kb in a single gel run. Thirdly, it resulted in faster electrophoretic gel run speed, and hence a lower required run time.

## Materials and Methods

A DNA ladder (1 Kb plus, Invitrogen) and 1% type I low-EEO agarose (Sigma) were used for all experiments. 10 cm long/5 mm thick gels were prepared with the same media as in the reservoir (see Fig. 3 and text below for a list of tested media). Horizontal gels (MGU-500, Del Mar, CA) were used for most experiments except when studying band compression (below). 750 mL reservoir medium was used for experiments devoted to conductance and heating, producing a 2 mm buffer overlay physically uniting the anodic and cathodic reservoirs. Conductivity of the medium was recorded as the measured current at constant voltage. Heat generation was implied from the increase in temperature measured in a reservoir. pH of the reservoirs was measured at the beginning and end of experiments. Constant voltage of 350 V for 40 minutes was employed with a safety lid in place.

For buffer exhaustion studies, reduced (600 mL) total volume of medium was used to obviate the buffer overlay, producing physical separation of the reservoir volumes while retaining the electrical path in series through the gel. Electrophoresis was terminated when current had decreased to 50% of its initial value.

Experiments measuring DNA compression were performed in vertical gels (SE 600, Hoefer, CA). Gels were 16 cm long/3 mm thick. Experiments were performed at constant voltage of 350 V and terminated upon maximal migration of the 200 bp band. The quality of separation of higher molecular weight bands was quantified using a compression ratio defined as the ratio of the distance separating the 200 bp and 1 kb bands relative to the distance separating the 4 kb and 12 kb bands (respectively, the areas of least and most compression in a conventional borate gel, when compared to an acetate gel). Ethidium bromide was absent during electrophoresis, and the DNA was post-stained.

All test media had 10 mM lithium cation and different anions chosen in consideration to their Lewis acid character. We tested borates, germanium hydroxide anion, germanium tetramethoxide anion, and phosphate, which were considered as strong Lewis acids due to their electron-deficient central atoms. We tested also acetate, salicylate, citrate, formate and propionate to represent weak Lewis acids. In general, we produced an alkaline solution containing 200 mM lithium ion; 4.79 g lithium hydroxide (reagent grade, Sigma-Aldrich, St. Louis, MO) was dissolved upon addition of deionized water to final volume of 1 liter while stirring at room temperature. Fifty ml of 200 mM lithium hydroxide was titrated to the desired pH (#476436, Pinnacle pH electrode, Nova Analytics, Woburn, MA) and brought to 1 liter volume (creating a 10 mM lithium ion concentration) by adding the acidic form of the desired anion (germanium hydroxide, germanium tetramethoxide, acetic acid, salicylic acid, citric acid, formic acid, or propionic acid) and deionized water while stirring at room temperature. Borate media at different pH were prepared by creating acidic and alkaline partner buffers at 10 mM lithium ion each. To 50 ml of the 200 mM lithium hydroxide was added boric acid powder (reagent grade, Sigma-Aldrich) and water while stirring at 75°C until the pH was below 6.0 and the volume was 1 liter; this acidic buffer was allowed to cool. A partner alkaline solution having 10 mM lithium ion was provided by diluting the alkaline 200 mM lithium ion solution or, alternately, diluting a 200 mM lithium borate/boric acid buffer (LB20-1®, Faster Better Media LLC, Hunt Valley, MD). The acidic and alkaline partner solutions (each having 10 mM lithium ion) were mixed at room temperature to achieve the desired pH of each test buffer. Likewise, phosphate buffers were made by combining 10 mM lithium ion solutions of lithium monobasic and lithium dibasic phosphate to the desired pH.

Gel elution was done by silica-based spin column (Cat # 28706, Qiagen), cellulose acetate spin filter (Spin-X, Costar) and glassmilk (Geneclean II, QBiogene), followed by Xba I restriction digestion.
